# Assessment of Fibrinogen-like 2 (FGL2) in Human Chronic Kidney Disease through Transcriptomics Data Analysis

**DOI:** 10.3390/biom13010089

**Published:** 2022-12-31

**Authors:** Sara Denicolò, Viji Nair, Johannes Leierer, Michael Rudnicki, Matthias Kretzler, Gert Mayer, Wenjun Ju, Paul Perco

**Affiliations:** 1 Department of Internal Medicine IV (Nephrology and Hypertension), Medical University Innsbruck, 6020 Innsbruck, Austria; 2 Department of Internal Medicine, University of Michigan, Ann Arbor, MI 48109, USA; 3 Department of Computational Medicine and Bioinformatics, University of Michigan, Ann Arbor, MI 48109, USA

**Keywords:** chronic kidney disease, renal fibrosis, FGL2, gene expression, outcome analysis

## Abstract

Fibrinogen-like 2 (FGL2) was recently found to be associated with fibrosis in a mouse model of kidney damage and was proposed as a potential therapeutic target in chronic kidney disease (CKD). We assessed the association of renal FGL2 mRNA expression with the disease outcome in two independent CKD cohorts (NEPTUNE and Innsbruck CKD cohort) using Kaplan Meier survival analysis. The regulation of FGL2 in kidney biopsies of CKD patients as compared to healthy controls was further assessed in 13 human CKD transcriptomics datasets. The FGL2 protein expression in human renal tissue sections was determined via immunohistochemistry. The regulators of FGL2 mRNA expression in renal tissue were identified in the co-expression and upstream regulator analysis of FGL2-positive renal cells via the use of single-cell RNA sequencing data from the kidney precision medicine project (KPMP). Higher renal FGL2 mRNA expression was positively associated with kidney fibrosis and negatively associated with eGFR. Renal FGL2 mRNA expression was upregulated in CKD as compared with healthy controls and associated with CKD progression in the Innsbruck CKD cohort (*p*-value = 0.0036) and NEPTUNE cohort (*p*-value = 0.0048). The highest abundance of FGL2 protein in renal tissue was detected in the thick ascending limb of the loop of Henle and macula densa, proximal tubular cells, as well as in glomerular endothelial cells. The upstream regulator analysis identified TNF, IL1B, IFNG, NFKB1, and SP1 as factors potentially inducing FGL2-co-expressed genes, whereas factors counterbalancing FGL2-co-expressed genes included GLI1, HNF1B, or PPARGC1A. In conclusion, renal FGL2 mRNA expression is elevated in human CKD, and higher FGL2 levels are associated with fibrosis and worse outcomes.

## 1. Introduction

Chronic kidney disease (CKD) is associated with considerable morbidity and mortality and was estimated to affect 9.1% (8.5 to 9.8) of the global population in 2017 [1]. Different etiologies and mechanisms are involved in the pathogenesis, but fibrosis is considered to be a common denominator across all etiologies. Complex processes leading to the enhanced formation and impaired degradation of the extracellular matrix, immune cell, especially macrophage, infiltrationand macrophage-to-myofibroblast as well as epithelial/endothelial-to-mesenchymal transition contribute to a progressive decline of kidney function and may finally lead to end-stage kidney disease (ESKD) [2,3]. A number of new therapeutic strategies, including antifibrotic drugs, were evaluated in preclinical studies and clinical trials within the last decade [4,5]. However, the available armamentarium to halt the progression of CKD, especially fibrosis, in clinical practice remains limited.

Fibrinogen-like 2 (FGL2) is a member of the fibrinogen superfamily that exists in a membrane-bound and soluble form [6,7]. While transmembrane FGL2 functions as a tissue prothrombinase and is mainly expressed on the surface of macrophages and endothelial and epithelial cells, soluble FGL2 functions as an immune modulator and effector protein of regulatory T-cells [6,7]. FGL2 was hypothesized to activate thrombin within the interstitial space resulting in fibrosis [8]. A study on the kidney biopsies of human acute allograft rejection showed a close association of FGL2 with fibrin depositions [9]. Recently, FGL2 was found to be markedly increased in a mouse model of kidney fibrosis induced by unilateral ureteral obstruction and in kidney biopsies of patients with CKD [10]. However, the same authors also found that renal fibrosis was increased in FGL2-deficient mice, proposing an upregulation of M2 macrophage polarization via the phosphorylation of STAT6 as a possible mechanism [10]. FGL2 might have multiple roles in the pathogenesis of fibrosis. Hitherto human data on FGL2 in chronic kidney disease are limited, and longitudinal studies are especially lacking.

In this study, we investigated the association of FGL2 gene expression with kidney function and longitudinal outcomes in two independent cohorts of patients with chronic kidney disease. We further compared the renal FGL2 gene expression in CKD patients and healthy controls, assessed the localization of FGL2 in kidney biopsies of CKD patients via immunohistochemistry and identified regulators of FGL2 gene expression in renal tissue using co-expression and upstream regulator analyses.

## 2. Results

### 2.1. Association of FGL2 Expression with CKD Progression in the Innsbruck CKD Cohort

A total of 37 stable and 26 progressive CKD patients with available gene expression and clinical data were used to assess the association of FGL2 expression with disease progression. The mean age was 47.2 years (±17.5), and 43% were female. In total, 5 patients had diabetic kidney disease (DKD), 7 had focal segmental glomerulosclerosis (FSGS), 7 had hypertensive kidney disease, 4 had IgA nephropathy (IgAN), 11 had lupus nephritis (LN), 10 had minimal change disease (MCD), 10 had membranous nephropathy (MN), 2 had membranoproliferative glomerulonephritis (MPGN), 5 had vasculitis, and 2 had etiologies that could not be further specified. The mean their baseline estimated glomerular filtration rate (eGFR) at baseline was 63.3 mL/min/1.73 m^2^ (±35.2), and the mean urine protein to creatinine ratio (UPCR) was 4.4 (±3.4) g/g creatinine. The mean follow-up time was 7.0 (±4.3) years. The baseline characteristics are shown in Table 1 and are subdivided into stable and progressive patients.

**Figure 1:**
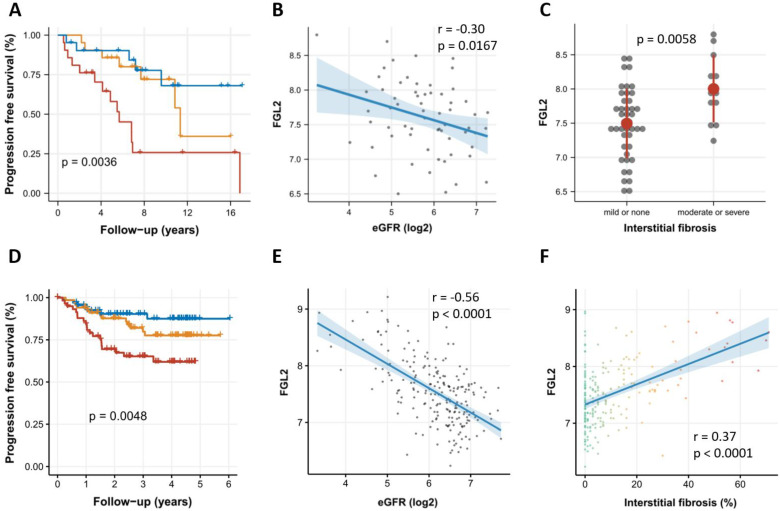
Association of FGL2 expression with renal outcomes in two independent CKD cohorts. FGL2 mRNA expression is significantly associated with the occurrence of the composite outcome (panel **A**), the baseline eGFR (panel **B**), and the degree of interstitial fibrosis (panel **C**) in the Innsbruck CKD cohort as well as in the NEPTUNE cohort (panels **D**–**F**). In panels (**A**,**D**), blue indicates the 1st (lowest), yellow the 2nd, and red the 3rd (highest) tercile of FGL2 mRNA expression.

The individuals were divided into terciles according to FGL2 mRNA expression. The patients with higher FGL2 mRNA expression levels were found to be more likely to have progressive CKD (*p* = 0.0036, log-rank test; Figure 1A). This finding did not change when age and sex, as well as the baseline eGFR and proteinuria, were added to a Cox regression model (crude HR: 3.47 [95% CI 1.43 to 8.45], adjusted for age and sex: HR 5.86 [95% CI 2.21 to 15.58], adjusted for age, sex, baseline eGFR, and proteinuria: 4.96 [95% CI 1.77 to 13.95]). FGL2 mRNA expression was negatively correlated with baseline eGFR (r = −0.30, *p* = 0.0167; Figure 1B) and higher in patients with moderate to severe interstitial fibrosis compared to patients with none or mild interstitial fibrosis (*p* = 0.0058, two-sided *t*-test; Figure 1C). Similar results were seen for tubular atrophy (*p* = 0.0028, two-sided *t*-test; Supplementary Figure S1A). FGL2 mRNA expression was not associated with glomerulosclerosis (*p* = 0.3064, two-sided *t*-test; Supplementary Figure S1B) or proteinuria (r = 0.018, *p* = 0.8927, Supplementary Figure S1C).

### 2.2. Association of FGL2 with CKD Progression in the NEPTUNE Cohort

FGL2 gene expression was assessed in 239 patients of the NEPTUNE cohort. The average age was 32.8 years (±22.8), and the mean eGFR value was 81 mL/min/1.73 m^2^ (±37.8). The mean follow-up time was 2.8 years (±1.5). The baseline characteristics of the NEPTUNE cohort are shown in Table 2.

The individuals were divided into terciles according to their FGL2 mRNA expression. The patients with higher FGL2 mRNA expression levels were also found to be more likely to have progressive CKD in the NEPTUNE cohort (*p* = 0.0048, log-rank test; Figure 1D). FGL2 mRNA expression in the NEPTUNE cohort was also negatively correlated with baseline eGFR (r = −0.56, *p* < 0.0001; Figure 1E) and positively correlated with interstitial fibrosis (r = 0.37, *p* < 0.0001; Figure 1F). FGL2 mRNA expression was associated with tubular atrophy (r = 0.38, *p* < 0.0001; Supplementary Figure S2A) but not with proteinuria (Supplementary Figure S2B).

### 2.3. FGL2 Is Significantly Higher Expressed in CKD

**Figure 2:**
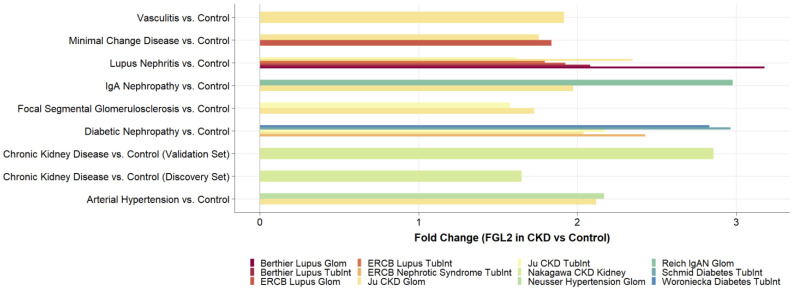
Upregulation of FGL2 mRNA expression in human CKD. FGL2 mRNA expression is significantly upregulated in renal tissue of CKD patients with different underlying CKD etiologies.

A total of 13 independent transcriptomics datasets containing 24 group comparisons between CKD patients and healthy controls were identified and extracted from NephroSeq (Supplementary Table S4). All transcriptomics analyses showed an overexpression of FGL2 in the kidneys of CKD patients (Figure 2). All but two analyses were statistically significant (Supplementary Table S4). The upregulation of FGL2 was consistent across different CKD etiologies (Figure 2).

### 2.4. FGL2 Protein Localization in CKD

**Figure 3:**
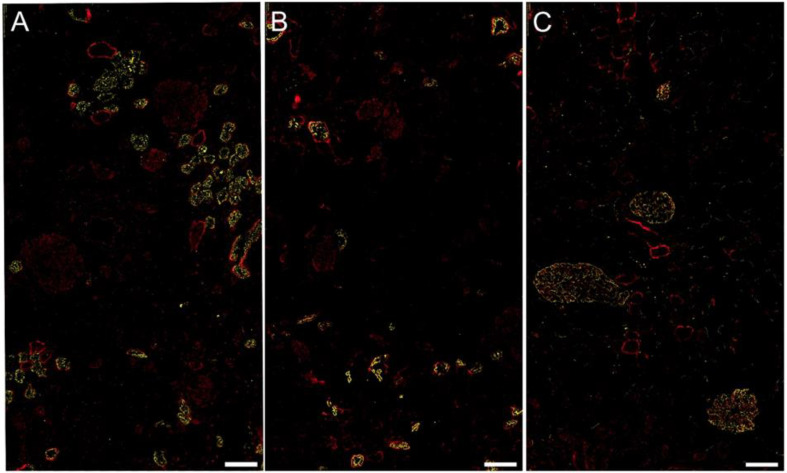
Protein localization of FGL2 in human renal tissue. Immunofluorescence double staining with the respective reference markers revealed the co-localization of FGL2 protein (red) with uromodulin (UMOD, yellow) in the distal tubule/thick ascending limb (TAL) cells, and macula densa (panel **A**) with aquaporin 2 (AQP2, yellow) in the proximal tubular cells (panel **B**) as well as with podocalyxin (PODXL, yellow) in glomerular cells (panel **C**). The scale bars indicate 100 µm.

FGL2 staining was mainly found in the distal tubules, thick ascending limbs, and macula densa. We also found positive staining in the proximal tubular cells as well as in glomerular cells (Figure 3).

### 2.5. Upstream Regulators of FGL2 Include TNF, IL1B, and IFNG

**Figure 4:**
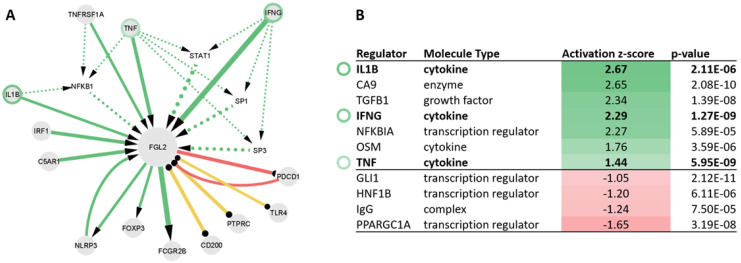
Mechanistic context of FGL2. Panel (**A**): genes identified in the literature co-citation analysis influencing FGL2 expression/activation, or being affected by FGL2 expression/activity. The green arrows indicate activation, the red arrows indicate inhibition, and the yellow connectors indicate associations. The intermediate transcription factors are connected with dotted edges. Panel (**B**): List of regulators identified in the IPA upstream regulator analysis to significantly influence the set of FGL2-associated genes based on the scRNAseq dataset in renal TAL cells. The regulators in bold font (IL1B, IFNG, and TNF) were also identified in the literature co-citation analysis, with all other regulators being considered novel regulators of FGL2 in renal TAL cells.

The literature co-citation analysis identified interferon gamma (IFNG), interleukin 1 beta (IL1B), tumor necrosis factor alpha (TNF), tumor necrosis factor alpha receptor 1 (TNFRSF1A), as well as interferon regulatory factor 1 (IRF1), complement component 5a receptor 1 (C5AR1), and NLR family pyrin domain containing 3 (NLRP3) as activators of FGL2. FGL2, on the other hand, was reported to induce forkhead box P3 (FOXP3), Fc gamma receptor IIb (FCGR2B), as well as NLRP3 (Figure 4A).

In order to find out whether these molecular interactions hold true also in kidney tissue, we examined the scRNAseq data in renal TAL cells in the KPMP dataset. We identified 109 genes showing significant upregulation with a fold-change of 1.15 or higher in FGL2+ TAL cells compared with FGL2− TAL cells. A total of 27 genes, on the other hand, were found to be significantly downregulated in FGL2+ TAL cells compared with FGL2− TAL cells. This FGL2 co-expressed gene set was used for the IPA upstream regulator analysis, and seven regulators were identified with a significant positive activation score, i.e., driving activation of the FGL2 co-expressed gene set. This set of activation factors, including IFNG, IL1B, and TNF, were also part of the literature-derived set of FGL2 activators (Figure 4B). Other activators of the FGL2 co-expressed gene set included carbonic anhydrase 9 (CA9), transforming growth factor beta 1 (TGFB1), NFKB inhibitor alpha (NFKBIA), and oncostatin M (OSM). Four regulators with negative activation z-scores were identified, namely GLI family zinc finger 1 (GLI1), HNF homeobox B (HNF1B), PPARG coactivator 1 alpha (PPARGC1A), as well as immunoglobulin G (IgG).

## 3. Discussion

In this study, we found that a higher FGL2 mRNA expression level in kidney biopsies of CKD patients was associated with lower eGFR and higher tubulointerstitial fibrosis at baseline as well as progression of CKD during the longitudinal observation period. The results were consistent across two independent CKD cohorts. FGL2 mRNA was also found to be significantly upregulated in renal tissue of CKD patients compared to samples from healthy controls based on the analysis of 13 different CKD transcriptomics datasets. The upregulation of FGL2 mRNA in CKD compared to healthy controls was observed regardless of the underlying CKD etiology.

The membrane-bound isoform of FGL2 acts as a direct prothrombinase that cleaves prothrombin to thrombin [11]. Thrombin is believed to contribute to interstitial fibrosis via the cleavage of fibrinogen to fibrin with consecutive fibrin deposition and fibroblast activation [8]. The FGL2 pathway might play a relevant role in interstitial thrombin generation [8]. It was previously reported that human kidney cells could synthesize prothrombin [12]. Hewitson and colleagues showed that thrombin acts as an in vitro agonist for kidney fibroblasts by increasing the cell population growth, the expression of procollagen α1, and the ability of fibroblasts to contract the collagen matrix [13]. The authors suggested that thrombin might contribute to the pathogenesis of kidney fibrosis via fibroblast activation and increased inflammation [13]. Similar to thrombin, fibrinogen was also found to stimulate kidney fibroblast proliferation in vitro directly [14]. Thrombin is also involved in the fibroblast-to-myofibroblast and epithelial-to-mesenchymal transitions, two processes linked to fibrosis development [15,16]. The dual staining of FGL2 and fibrin in the kidney biopsies of human acute allograft rejections showed a spatial proximity of both proteins in the glomerulus, microvascular vessels, within kidney tubules, and on infiltrating mononuclear cells [9]. In a murine model of diabetic kidney disease, FGL2 was increased in the glomerular and tubulointerstitial capillaries and associated with microthrombosis [17]. Based on these previous findings, a pro-fibrotic effect of the membrane-bound form of FGL2 seems plausible. This is in line with our results showing increased interstitial fibrosis and tubular atrophy, a lower eGFR, as well as a worse CKD prognosis with a higher FGL2 mRNA expression level in kidney biopsies. FGL2 mRNA was upregulated in CKD compared to healthy controls regardless of the underlying etiology. This is in line with tubulointerstitial fibrosis being a shared pathological feature of all CKD entities. FGL2 might primarily act in the tubulointerstitial space rather than the glomerulus, as UPCR and glomerulosclerosis were not associated with FGL2 mRNA expression.

Soluble FGL2 is secreted by immune cells, especially regulatory T cells, and has an immunoregulatory function. It lacks the prothrombinase function of its membrane-bound isoform [18]. Shalev and colleagues found that mice with a targeted deletion of FGL2 as well as wild-type mice reconstituted with FGL2−/− bone marrow developed autoimmune glomerulonephritis with increasing age [18]. Regulatory T cell activity was significantly impaired in these mouse models, and antibodies to FGL2 inhibited regulatory T cell activity in an in vitro model [18]. Soluble FGL2 was suggested to counteract pro-inflammatory stimuli and regulate the resolution of inflammation [19]. The shedding of soluble FGL2 from the cell membrane seems to play an important role in this process [19].

Data on the therapeutic targeting of FGL2 are conflicting. The suppression, as well as over-expression of FGL2, were both associated with longer graft survival in experimental models of heart transplantation [9,20,21]. Wu and colleagues found that FGL2 deficiency aggravated kidney fibrosis by the p-STAT6-dependent upregulation of M2 macrophage polarization in a murine model [10]. Soluble FGL2 induced the apoptosis of tubular epithelial cells in vitro by upregulating pro-apoptotic genes [22,23]. However, it is not clear whether this contributes to kidney damage or tissue repair [22,23]. It is possible that the membrane-bound isoform of FGL2 exhibits pro-fibrotic and pro-inflammatory effects on the kidney, while its soluble isoform attempts to attenuate inflammation and fibrosis and repair tissue damage. In a study on sepsis, patients who did not survive had decreased plasma levels of soluble FGL2 and upregulated membrane-bound FGL2 on leukocytes compared to patients who survived [19]. Further, an earlier increase in membrane-bound FGL2 and lower soluble FGL2 was associated with a worse outcome [19]. Increased soluble FGL2 in kidney tissue might be the expression of an insufficient compensatory mechanism in response to kidney damage. There might be a fragile balance between both isoforms of FGL2, as there is between tissue repair and fibrosis.

By analyzing datasets in NephroSeq, we found that FGL2 mRNA was markedly increased in the biopsies of CKD patients compared to healthy controls. This is in line with two other studies showing increased FGL2 protein expression in the renal tissue of patients with CKD and patients with acute allograft rejection [9,10].

Based on the literature co-citation analyses, IFNG, IL1B, TNF, TNFRSF1A, IRF1, C5AR1, and NLRP3 were identified as activators of FGL2. All proteins play a pro-inflammatory role in the regulation of inflammation [24,25]. IFNG, IL1B, and TNF were also identified as activating factors of FGL2 in TAL cells. Almost all other identified activating factors of FGL2 in TAL cells (TGFB1, NFKB, NFKBIA, and OSM) are also known to modulate inflammation [24,25]. Molecules that showed negative activation z-scores with regard to FGL2 in TAL cells were mostly transcription factors or transcription regulators (GLI1, HNF1B, and PPARGC1A) [24,25]. Based on these analyses, the pro-inflammatory environment in CKD might stimulate FGL2 expression in the kidney. The downregulation of activating factors or the upregulation of negative regulators might pose a therapeutic strategy to counterbalance the FGL2 co-expression signature in TAL cells.

This study has limitations that need to be considered when interpreting our findings. First, it is a retrospective analysis, and a significant part of this study consists of the re-analyses of existing datasets. Second, all patients in both cohorts had proteinuria. Thus, we could not determine whether the same results apply to CKD patients without proteinuria. Third, we could not discriminate between both isoforms of FGL2 as they are formed by post-transcriptional modifications, and there are no commercially available antibodies for targeting one of the two isoforms.

In this study, we analyzed FGL2 in the context of human CKD. The consistency of our results across a number of data sets suggests an important role of FGL2 in human CKD and tubulointerstitial fibrosis.

## 4. Materials and Methods

### 4.1. FGL2 Outcome Analysis in the Innsbruck CKD Cohort

We determined the association of FGL2 gene expression in kidney biopsies with disease progression by re-analyzing a retrospective cohort of 63 patients with various CKD diagnoses [26,27]. Patients with a minimum follow-up time of six months who reached ESKD or experienced a 40% reduction in eGFR during the follow-up period were defined as “progressive”. Patients who did not develop ESKD nor experienced a 40% reduction in their baseline eGFR during their follow-up with a minimum available follow-up time of twelve months were defined as “stable”. The clinical data from the last available follow-up visits were recorded in the stable patients. The modification of diet in renal disease study (MDRD) formula was used to calculate the eGFR values. Proteinuria was estimated by calculating the UPCR. Information on the degree of histological damage recorded by a pathologist was available for 55 patients. Interstitial fibrosis and tubular atrophy were defined semi-quantitatively as absent (“none”), “mild”, “moderate”, or “severe” based on routine pathology reports. Glomerulosclerosis was described as being “absent” or “present”. The continuous variables were described as the means and standard deviations or as the median and 25% and 75% quantiles, as appropriate. The discrete variables were described as numbers and relative frequencies. Pearson correlation coefficients were used to describe the relationship between FGL2 mRNA expression and eGFR and proteinuria at baseline. Two-sided *t*-tests were used to describe the differences in FGL2 mRNA expression by the degree of interstitial fibrosis, tubular atrophy, and glomerulosclerosis. The progression of CKD by FGL2 mRNA expression was analyzed with the Kaplan Meier method and log-rank test.

### 4.2. FGL2 Outcome Analysis in the NEPTUNE Cohort

The association of FGL2 gene expression with disease progression was assessed in samples from the NEPTUNE cohort [28]. NEPTUNE was a multicenter prospective cohort study of patients with proteinuric glomerular disease, for which their comprehensive clinical and molecular phenotyping data was collected at 21 sites at the time of the study [29]. A listing of all NEPTUNE members is available in Supplementary Data S1.

FGL2 correlations to eGFR and UPCR, as well as to the two histological parameters degree of interstitial fibrosis and degree of tubular atrophy, were determined using Spearman’s rank correlation coefficient. The degree of interstitial fibrosis and tubular atrophy was reported as 0–100% of the cortex involved and was assessed by at least five independent pathologists and provided as the average percentage of the obtained scores [28,30]. The association with disease progression was determined in a time-to-event analysis using a composite endpoint of ESKD or a 40% reduction in the baseline eGFR.

### 4.3. FGL2 mRNA Expression Analysis in CKD Patients as Compared with Healthy Controls

Gene expression data from NephroSeq (www.nephroseq.org (accessed on 24 November 2022)) were retrieved to compare the FGL2 mRNA expression levels in the renal cortex and tubulointerstitial compartments between CKD patients and healthy controls in the data from Berthier et al. [31], Ju et al. [32], Nakagawa et al. [33], Neusser et al. [34], Reich et al. [35], Schmid et al. [36], Woroniecka et al. [37], Hodgin et al. [38], as well as from the ERCB cohort [39].

NephroSeq was searched applying the following filters: analysis type: „disease vs. control analyses”; tissue type: „glomeruli“, „tubulointerstitium“, „other kidney part“; organism: „human“; fold change threshold: 1.5. Data of kidney transplant biopsies and comparisons between different CKD etiologies were excluded. The data were extracted on 2nd September 2021.

### 4.4. FGL2 Protein Staining in Human CKD Tissue Samples

Five μm-thick sections were cut from fresh frozen tissue on a cryostat (Leica, Vienna, Austria) at −20 °C and mounted on poly-l-lysine-coated slides. The sections were blocked with PBS containing 20% goat or fetal calf serum at room temperature for one hour. Primary antibodies, as listed in Supplementary Table S1, were diluted in PBS containing 2% serum and applied to sections after removing the blocking solution without additional washing and incubated at 4 °C overnight. Double stainings with antibodies directed against specific regions of the kidney (UMOD for the distal tubule, PODXL for the glomeruli, and AQP2 for the proximal tubule) were performed to specify the location of FGL2.

After three washes with PBS, the sections were incubated with the secondary antibodies, as listed in Supplementary Table S2, for one hour at room temperature. In the control experiments, no immunoreactivity could be detected when the primary antibody was omitted.

### 4.5. Literature Co-Annotation Analysis of FGL2

We performed a literature co-citation analysis to find potential regulators of FGL2 as well as downstream targets. We downloaded NCBI’s gene2pubmed file on 27th September 2021, and extracted all publications listing FGL2, and, in a second step, all additional genes associated with the set of publications on FGL2 were extracted. We considered the gene2pubmed entries with taxonomy IDs (taxids) for “Homo sapiens” (taxid:9606), “Mus musculus” (taxid:10090), and “Rattus norvegicus” (taxid:10116). Publications holding genes co-mentioned with FGL2 in at least two publications were manually checked, and the associations with FGL2 were extracted from the respective publications. Cytoscape version 3.9.0 was used to visualize the associations with FGL2.

### 4.6. Single Cell RNA Sequencing (scRNA-seq) Analysis

ScRNAseq data from the kidney biopsies of 10 participants diagnosed with DKD were downloaded from the kidney precision medicine project (KPMP) kidney tissue atlas and processed according to the KPMP single-cell protocol as previously described [40]. The basic characteristics of the included patients are demonstrated in Supplementary Table S3. Tissue processing and single-cell isolation were performed according to our published protocol [41]. Following the standard analytical protocol using the Seurat R package, including SCTransform, the dimensionality reduction principal component analysis (PCA) and UMAP, and standard unsupervised clustering, 25 cell clusters were mapped using known marker genes. We specifically focused on the thick ascending limb (TAL) cell cluster, as FGL2 was found to be abundant in this renal compartment based on the protein staining experiments.

TAL cells showing FGL2 mRNA expression greater than zero were defined as FGL2 positive (+) cells. The remaining cells of the TAL cell cluster were grouped as FGL2 negative (−). Differentially expressed genes between FGL2 (+) vs. FGL2 (−) TAL cells were identified using the FINDMARKER function implemented in the Seurat R package. All genes showing Bonferroni-adjusted *p*-values < 0.05 were selected for the downstream analysis.

### 4.7. Ingenuity Upstream Regulator Analysis

An upstream regulator analysis (URA) was performed as implemented in Ingenuity Pathways Analysis (IPA) software on the FGL2 (+) TAL co-regulated genes. This approach identifies potential upstream regulators, such as transcriptional factors, growth factors, and cytokines, which could impact the expression pattern of the gene set being studied.

We considered all upstream regulators of the FGL2-associated gene set as relevant that had no “Bias”-flag showing z-scores > 1 and *p*-values < 0.0001.

## Figures and Tables

**Table 1:** Patient characteristics of the Innsbruck CKD cohort.

Baseline Characteristics	Cohort (N = 63)	Progressive (N = 26)	Stable (N = 37)
Age (years)	47.2 ± 17.5	55.9 ± 14.4	41.1 ± 17.0
eGFR (mL/min/1.73 m^2^)	63.3 ± 35.2	50.1 ± 32.5	72.5 ± 34.5
UPCR (g/g)	4.4 ± 3.4	4.2 ± 2.7	4.5 ± 3.8
Follow-up time (years)	7.0 ± 4.3	5.2 ± 3.9	8.2 ± 4.2
Female gender (n (%))	27 (43)	7 (27)	20 (54)

The key clinical parameters are provided for the whole cohort as well as for the stable and progressive subgroups. The mean values plus standard deviations are provided for the continuous parameters, and the number and relative frequencies are provided for the discrete variables. eGFR = estimated glomerular filtration rate; UPCR = urine protein to creatinine ratio.

**Table 2:** Patient characteristics of the NEPTUNE cohort.

Baseline Characteristics	Cohort (N = 239)	FSGS (N = 67)	MCD (N = 55)	MN (N = 47)	Other Diseases (N = 70)
Age (years)	32.8 ± 22.8	33.9 ± 22.3	20.6 ± 20.8	47.4 ± 18.3	31.6 ± 22
eGFR (mL/min/1.73 m^2^)	81.0 ± 37.8	75.6 ± 34.7	104.4 ± 40.7	79.1 ± 27.6	69.4 ± 37.7
UPCR (g/g)	4.5 ± 4.5	4.2 ± 5.2	3.8 ± 4.1	6.1 ± 4.0	4.0 ± 4.3
Follow-up time (years)	2.8 ± 1.5	2.8 ± 1.8	2.5 ± 1.9	2.2 ± 1.9	1.8 ± 1.4
Female gender (n (%))	86 (36)	21 (31)	20 (36)	19 (40)	26 (37)

Key clinical parameters of the NEPTUNE cohort are provided for the whole cohort as well as for the different diagnosis subgroups. The mean values plus standard deviations are provided for the continuous parameters, and the number and relative frequencies are provided for the discrete variables. eGFR = estimated glomerular filtration rate; UPCR = urine protein to creatinine ratio; FSGS = focal segmental glomerulosclerosis; MCD = minimal change disease; MN = membranous nephropathy.

## Data Availability

The gene expression data are stored in NephroSeq and KPMP as described in the materials and methods section. All other data are included in the main manuscript or the supplementary materials.

## Supplementary Materials

The following supporting information can be downloaded at: https://www.mdpi.com/article/10.3390/biom13010089/s1, Figure S1: Association of FGL2 expression with baseline parameters in the Innsbruck CKD cohort. Figure S2: Association of FGL2 expression with baseline parameters in the NEPTUNE cohort. Table S1: List of primary antibodies used in immunostaining experiments. Table S2: List of secondary antibodies used in immunostaining experiments. Table S3: Baseline characteristics of KPMP participants included in scRNASeq analysis. Table S4: Analyses of FGL2 expression in transcriptomics datasets available through NephroSeq. Data S1: Members of the Nephrotic Syndrome Study Network (NEPTUNE).

## References

[1] Bikbov B., Purcell C.A., Levey A.S., Smith M., Abdoli A., Abebe M., Adebayo O.M., Afarideh M., Agarwal S.K., Agudelo-Botero M. (2020). Global, regional, and national burden of chronic kidney disease, 1990–2017: A systematic analysis for the Global Burden of Disease Study 2017. Lancet.

[2] Meng X.-M., Nikolic-Paterson D.J., Lan H.Y. (2014). Inflammatory processes in renal fibrosis. Nat. Rev. Nephrol..

[3] Tang P.M.K., Nikolic-Paterson D.J., Lan H.Y. (2019). Macrophages: Versatile players in renal inflammation and fibrosis. Nat. Rev. Nephrol..

[4] Tampe D., Zeisberg M. (2014). Potential approaches to reverse or repair renal fibrosis. Nat. Rev. Nephrol..

[5] Klinkhammer B.M., Goldschmeding R., Floege J., Boor P. (2017). Treatment of Renal Fibrosis—Turning Challenges into Opportunities. Adv. Chronic Kidney Dis..

[6] Yang G. (2013). Physiological functions and clinical implications of fibrinogen-like 2: A review. World J. Clin. Infect. Dis..

[7] Hu J., Yan J., Rao G., Latha K., Overwijk W.W., Heimberger A.B., Li S. (2016). The Duality of Fgl2—Secreted Immune Checkpoint Regulator Versus Membrane-Associated Procoagulant: Therapeutic Potential and Implications. Int. Rev. Immunol..

[8] de Ridder G.G., Lundblad R.L., Pizzo S.V. (2016). Actions of thrombin in the interstitium. J. Thromb. Haemost..

[9] Ning Q., Sun Y., Han M., Zhang L., Zhu C., Zhang W., Guo H., Li J., Yan W., Gong F. (2005). Role of Fibrinogen-Like Protein 2 Prothrombinase/Fibroleukin in Experimental and Human Allograft Rejection. J. Immunol..

[10] Wu S., Li M., Xu F., Li G.-Q., Han B., He X.-D., Li S.-J., He Q.-H., Lai X.-Y., Zhou S. (2020). Fibrinogen-like protein 2 deficiency aggravates renal fibrosis by facilitating macrophage polarization. Biomed. Pharmacother..

[11] Yuwaraj S., Ding J.W., Liu M., Marsden P.A., Levy G.A. (2001). Genomic characterization, localization, and functional expression of FGL2, the human gene encoding fibroleukin: A novel human procoagulant. Genomics.

[12] Stenberg L.M., Brown M.A., Nilsson E., Ljungberg O., Stenflo J. (2001). A functional prothrombin gene product is synthesized by human kidney cells. Biochem. Biophys. Res. Commun..

[13] Hewitson T.D., Martic M., Kelynack K.J., Pagel C.N., Mackie E.J., Becker G.J. (2005). Thrombin is a pro-fibrotic factor for rat renal fibroblasts in vitro. Nephron. Exp. Nephrol..

[14] Sörensen I., Susnik N., Inhester T., Degen J.L., Melk A., Haller H., Schmitt R. (2011). Fibrinogen, acting as a mitogen for tubulointerstitial fibroblasts, promotes renal fibrosis. Kidney Int..

[15] Wygrecka M., Didiasova M., Berscheid S., Piskulak K., Taborski B., Zakrzewicz D., Kwapiszewska G., Preissner K.T., Markart P. (2013). Protease-activated receptors (PAR)-1 and -3 drive epithelial-mesenchymal transition of alveolar epithelial cells—Potential role in lung fibrosis. Thromb. Haemost..

[16] Bogatkevich G.S., Tourkina E., Silver R.M., Ludwicka-Bradley A. (2001). Thrombin differentiates normal lung fibroblasts to a myofibroblast phenotype via the proteolytically activated receptor-1 and a protein kinase C-dependent pathway. J. Biol. Chem..

[17] Su G., Liu K., Wang Y., Wang J., Li X., Li W., Liao Y., Wang Z. (2011). Fibrinogen-like protein 2 expression correlates with microthrombosis in rats with type 2 diabetic nephropathy. J. Biomed. Res..

[18] Shalev I., Liu H., Koscik C., Bartczak A., Javadi M., Wong K.M., Maknojia A., He W., Liu M.F., Diao J. (2008). Targeted deletion of fgl2 leads to impaired regulatory T cell activity and development of autoimmune glomerulonephritis. J. Immunol..

[19] Zhou Y., Lei J., Xie Q., Wu L., Jin S., Guo B., Wang X., Yan G., Zhang Q., Zhao H. (2019). Fibrinogen-like protein 2 controls sepsis catabasis by interacting with resolvin Dp5. Sci. Adv..

[20] Bézie S., Picarda E., Tesson L., Renaudin K., Durand J., Ménoret S., Mérieau E., Chiffoleau E., Guillonneau C., Caron L. (2015). Fibrinogen-Like Protein 2/Fibroleukin Induces Long-Term Allograft Survival in a Rat Model through Regulatory B Cells. PLoS ONE.

[21] Bézie S., Ménoret S., Tesson L., Li X.-L., Usal C., Anegon I., Caron L. (2011). Immunosuppressive role of fibrinogen-like protein 2 (FGL2) in CD8+regulatory T cells-mediated long-term graft survival. J. Transl. Med..

[22] Zhao Z., Yang C., Li L., Zhao T., Wang L., Rong R., Yang B., Xu M., Zhu T. (2014). Increased peripheral and local soluble FGL2 in the recovery of renal ischemia reperfusion injury in a porcine kidney auto-transplantation model. J. Transl. Med..

[23] Zhao Z., Yang C., Wang L., Li L., Zhao T., Hu L., Rong R., Xu M., Zhu T. (2014). The regulatory T cell effector soluble fibrinogen-like protein 2 induces tubular epithelial cell apoptosis in renal transplantation. Exp. Biol. Med..

[24] Uhlén M., Fagerberg L., Hallström B.M., Lindskog C., Oksvold P., Mardinoglu A., Sivertsson Å., Kampf C., Sjöstedt E., Asplund A. (2015). Proteomics. Tissue-based map of the human proteome. Science.

[25] The Human Protein Atlas. https://www.proteinatlas.org/.

[26] Rudnicki M., Perco P., Haene D.B., Leierer J., Heinzel A., Mühlberger I., Schweibert N., Sunzenauer J., Regele H., Kronbichler A. (2016). Renal microRNA- and RNA-profiles in progressive chronic kidney disease. Eur. J. Clin. Investig..

[27] Perco P., Ju W., Kerschbaum J., Leierer J., Menon R., Zhu C., Kretzler M., Mayer G., Rudnicki M., Nephrotic Syndrome Study Network (NEPTUNE) (2019). Identification of dicarbonyl and L-xylulose reductase as a therapeutic target in human chronic kidney disease. JCI Insight.

[28] Mariani L.H., Martini S., Barisoni L., Canetta P.A., Troost J.P., Hodgin J.B., Palmer M., Rosenberg A.Z., Lemley K.V., Chien H.-P. (2018). Interstitial fibrosis scored on whole-slide digital imaging of kidney biopsies is a predictor of outcome in proteinuric glomerulopathies. Nephrol. Dial. Transpl..

[29] Gadegbeku C.A., Gipson D.S., Holzman L.B., Ojo A.O., Song P.X.K., Barisoni L., Sampson M.G., Kopp J.B., Lemley K.V., Nelson P.J. (2013). Design of the Nephrotic Syndrome Study Network (NEPTUNE) to evaluate primary glomerular nephropathy by a multidisciplinary approach. Kidney Int..

[30] Barisoni L., Gimpel C., Kain R., Laurinavicius A., Bueno G., Zeng C., Liu Z., Schaefer F., Kretzler M., Holzman L.B. (2017). Digital pathology imaging as a novel platform for standardization and globalization of quantitative nephropathology. Clin. Kidney J..

[31] Berthier C.C., Bethunaickan R., Gonzalez-Rivera T., Nair V., Ramanujam M., Zhang W., Bottinger E.P., Segerer S., Lindenmeyer M., Cohen C.D. (2012). Cross-Species Transcriptional Network Analysis Defines Shared Inflammatory Responses in Murine and Human Lupus Nephritis. J. Immunol..

[32] Ju W., Greene C.S., Eichinger F., Nair V., Hodgin J.B., Bitzer M., Lee Y.-S., Zhu Q., Kehata M., Li M. (2013). Defining cell-type specificity at the transcriptional level in human disease. Genome Res..

[33] Nakagawa S., Nishihara K., Miyata H., Shinke H., Tomita E., Kajiwara M., Matsubara T., Iehara N., Igarashi Y., Yamada H. (2015). Molecular Markers of Tubulointerstitial Fibrosis and Tubular Cell Damage in Patients with Chronic Kidney Disease. PLoS ONE.

[34] Neusser M.A., Lindenmeyer M.T., Moll A.G., Segerer S., Edenhofer I., Sen K., Stiehl D.P., Kretzler M., Gröne H.J., Schlöndorff D. (2010). Human nephrosclerosis triggers a hypoxia-related glomerulopathy. Am. J. Pathol..

[35] Reich H.N., Tritchler D., Cattran D.C., Herzenberg A.M., Eichinger F., Boucherot A., Henger A., Berthier C.C., Nair V., Cohen C.D. (2010). A molecular signature of proteinuria in glomerulonephritis. PLoS ONE.

[36] Schmid H., Boucherot A., Yasuda Y., Henger A., Brunner B., Eichinger F., Nitsche A., Kiss E., Bleich M., Gröne H.J. (2006). Modular activation of nuclear factor-κB transcriptional programs in human diabetic nephropathy. Diabetes.

[37] Woroniecka K.I., Park A.S.D., Mohtat D., Thomas D.B., Pullman J.M., Susztak K. (2011). Transcriptome analysis of human diabetic kidney disease. Diabetes.

[38] Hodgin J.B., Borczuk A.C., Nasr S.H., Markowitz G.S., Nair V., Martini S., Eichinger F., Vining C., Berthier C.C., Kretzler M. (2010). A Molecular Profile of Focal Segmental Glomerulosclerosis from Formalin-Fixed, Paraffin-Embedded Tissue. Am. J. Pathol..

[39] Ju W., Nair V., Smith S., Zhu L., Shedden K., Song P.X.K., Mariani L.H., Eichinger F.H., Berthier C.C., Randolph A. (2015). Tissue transcriptome-driven identification of epidermal growth factor as a chronic kidney disease biomarker. Sci. Transl. Med..

[40] Menon R., Otto E.A., Sealfon R., Nair V., Wong A.K., Theesfeld C.L., Chen X., Wang Y., Boppana A.S., Luo J. (2020). SARS-CoV-2 receptor networks in diabetic and COVID-19-associated kidney disease. Kidney Int..

[41] Menez S., Ju W., Menon R., Moledina D.G., Thiessen Philbrook H., McArthur E., Jia Y., Obeid W., Mansour S.G., Koyner J.L. (2021). Urinary EGF and MCP-1 and risk of CKD after cardiac surgery. JCI Insight.

