# Supplementary material for: Assessment of Fibrinogen-like 2 (FGL2) in Human Chronic Kidney Disease through Transcriptomics Data Analysis
Source: Biomolecules. 2022 Dec 31;13(1):89. doi: 10.3390/biom13010089 (PMC9855364; doi:10.3390/biom13010089)
Supplement: Supplementary file 1 [file biomolecules-13-00089-s001.zip › biomolecules-2089732-supplementary.pdf]

SUPPLEMENTARY MATERIAL

TABLE OF CONTENT

Supplementary Figure S1 – Association of FGL2 expression with baseline parameters in the Innsbruck CKD cohort 2

Supplementary Figure S2 – Association of FGL2 expression with baseline parameters in the NEPTUNE cohort 3

Supplementary Table S1 – List of primary antibodies used in immunostaining experiments 4

Supplementary Table S2 – List of secondary antibodies used in immunostaining experiments.. 5

Supplementary Table S3 – Baseline characteristics of KPMP participants included in scRNASeq analysis. 6

Supplementary Table S4 – Analyses of FGL2 expression in transcriptomics datasets available through NephroSeq 7

Supplementary Data S1 – Members of the Nephrotic Syndrome Study Network (NEPTUNE) 9

Supplementary Figure S1 – Association of FGL2 expression with baseline parameters in the Innsbruck CKD cohort

FGL2 expression is significantly associated with tubular atrophy (*Panel A*) but not with the degree of glomerulosclerosis (*Panel B*) and proteinuria (*Panel C*).

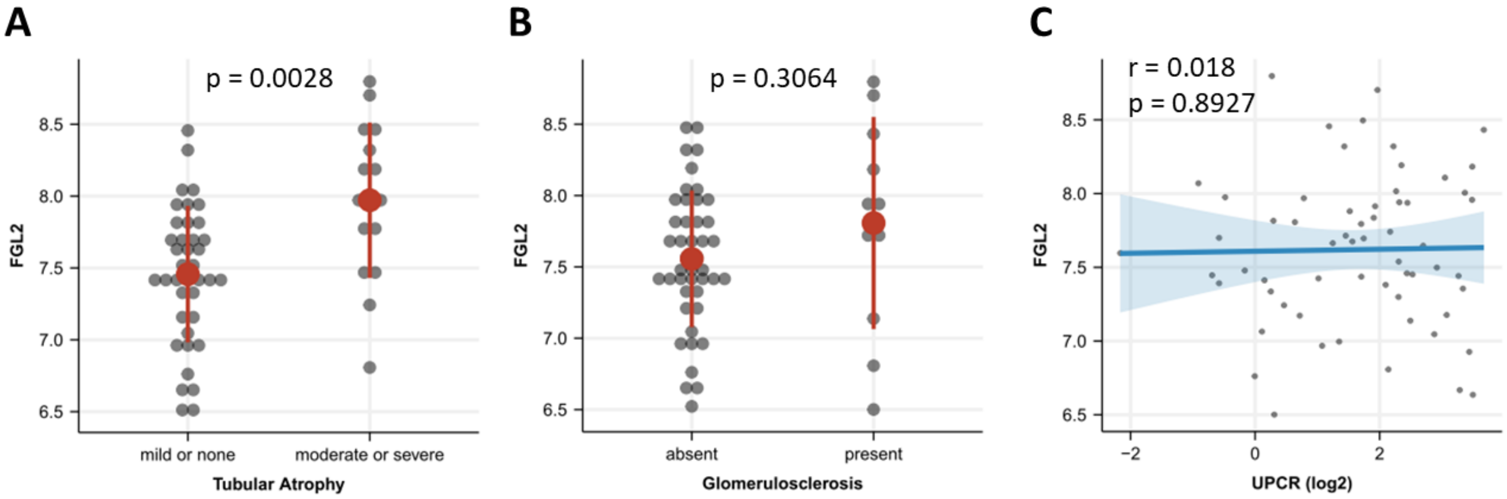

Supplementary Figure S2 – Association of FGL2 expression with baseline parameters in the NEPTUNE cohort

FGL2 expression is significantly associated with tubular atrophy (*Panel A*) but not with baseline proteinuria (*Panel B*)

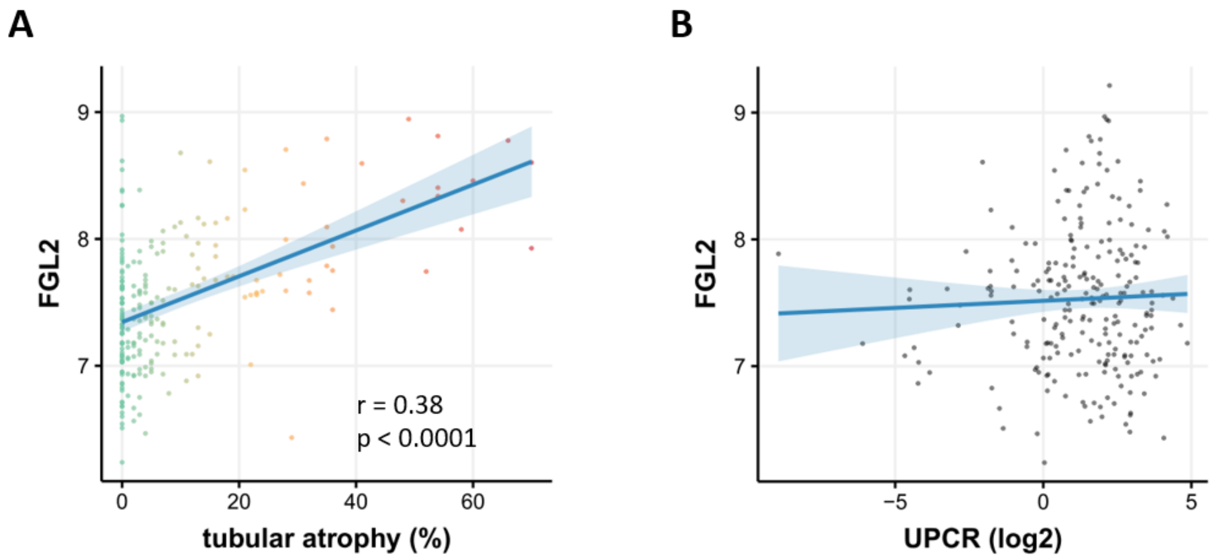

Supplementary Table S1 – List of primary antibodies used in immunostaining experiments

Protein	Reactivity	Source	Ig-Class	Company	Order number	Dilution
FGL2	Human	Mouse	monoclonal	Abnova	H00010875-M01	1:1000
AQP2	Human	Rabbit	polyclonal	Sigma-Aldrich/Merck	HPA046834	1:1000
PODXL	Human	Rabbit	polyclonal	Sigma-Aldrich/Merck	HPA045507	1:1000
UMOD	Human	Rabbit	polyclonal	Sigma-Aldrich/Merck	HPA043420	1:1000

Supplementary Table S2 – List of secondary antibodies used in immunostaining experiments

Label	Source	directed against	Ig-Class	Company	Cat.No.	Dilution
Alexa Fluor+ 555	Donkey	Anti-Rabbit	IgG	Thermo Fisher	A32794	1:2000
Alexa Fluor+ 647	Donkey	anti-Mouse	IgG	Thermo Fisher	A32787	1:2000

Supplementary Table S3 – Baseline characteristics of KPMP participants included in scRNASeq analysis.

Sample	Gender	Age	Race	Baseline eGFR (ml/min/1.73m2)	Proteinuria (mg)	A1c (%)	Albuminuria (mg)	Diabetes History	Diabetes Duration	Hypertension History	Hypertension Duration	On RAAS Blockade
S1	Female	62	White	40-49		> 8.5	500-999mg	Yes	30-34	Yes	30-34	Yes
S2	Male	64	White	30-39	< 150mg	> 8.5	< 30mg	Yes	0-4	No		No
S3	Female	66	Black or African-American	80-89	500-999mg		500-999mg	Yes	5-9	Yes	30-34	Yes
S4	Male	71		40-49				Yes	10-14	Yes	0-4	No
S5	Female	34	White	30-39	>= 1000mg	6.5-7.49		Yes	20-24	Yes	5-9	Yes
S6	Female	63	Black or African-American	110-119	< 150mg	6.5-7.49	30-299mg	Yes	5-9	Yes	20-24	Yes
S7	Female	33	White	100-109	500-999mg		500-999mg	Yes	20-24	Yes	0-4	Yes
S8	Male	74	White	40-49	150mg-499mg	6.5-7.49	< 30mg	Yes	25-29	Yes	25-29	Yes
S9	Female	77	White	60-69	>= 1000mg	6.5-7.49	>= 1000mg	Yes	10-14	Yes	10-14	Yes
S10	Female	67	White,Other	20-29	>= 1000mg	< 6.5	>= 1000mg	Yes	20-24	Yes	0-4	No

Supplementary Table S4 – Analyses of FGL2 expression in transcriptomics datasets available through NephroSeq

Dataset	Analysis	Analysis Synopsis	Analysis Type	p-Value	Fold Change	Data Type	Reporter
Berthier Lupus Glom	Lupus Nephritis vs. Healthy Living Donor	Disease vs. Control	over expression	< 0.0001	3.18	mRNA	204834_at
Berthier Lupus TubInt	Lupus Nephritis vs. Healthy Living Donor	Disease vs. Control	over expression	< 0.0001	2.08	mRNA	204834_at
ERCB Lupus Glom	Minimal Change Disease vs. Healthy Living Donor	Disease vs. Control	over expression	0.02900	1.84	mRNA	ENSG00000127951
ERCB Lupus Glom	Lupus Nephritis vs. Healthy Living Donor	Disease vs. Control	over expression	0.01100	1.92	mRNA	ENSG00000127951
ERCB Lupus TubInt	Lupus Nephritis vs. Healthy Living Donor	Disease vs. Control	over expression	0.03300	1.80	mRNA	ENSG00000127951
ERCB Nephrotic Syndrome TubInt	Diabetic Nephropathy vs. Healthy Living Donor	Disease vs. Control	over expression	0.00014	2.43	mRNA	ENSG00000127951
Hodgin FSGS Glom	Focal Segmental Glomerulosclerosis vs. Normal Kidney	Disease vs. Control	over expression	0.26000	1.55	mRNA	Hs.296276.0.A1_3p_at
Hodgin FSGS Glom	Minimal Change Disease vs. Normal Kidney	Disease vs. Control	over expression	0.07300	2.37	mRNA	g5730074_3p_at
Ju CKD Glom	Focal Segmental Glomerulosclerosis vs. Healthy Living Donor	Disease vs. Control	over expression	< 0.0001	1.73	mRNA	10875
Ju CKD Glom	Minimal Change Disease vs. Healthy Living Donor	Disease vs. Control	over expression	0.00200	1.76	mRNA	10875
Ju CKD Glom	Vasculitis vs. Healthy Living Donor	Disease vs. Control	over expression	< 0.0001	1.92	mRNA	10875
Ju CKD Glom	IgA Nephropathy vs. Healthy Living Donor	Disease vs. Control	over expression	< 0.0001	1.97	mRNA	10875

Ju CKD Glom	Diabetic Nephropathy vs. Healthy Living Donor	Disease vs. Control	over expression	< 0.0001	2.04	mRNA	10875
Ju CKD Glom	Arterial Hypertension vs. Healthy Living Donor	Disease vs. Control	over expression	< 0.0001	2.12	mRNA	10875
Ju CKD Glom	Lupus Nephritis vs. Healthy Living Donor	Disease vs. Control	over expression	< 0.0001	2.35	mRNA	10875
Ju CKD TubInt	Focal Segmental Glomerulosclerosis vs. Healthy Living Donor	Disease vs. Control	over expression	0.00096	1.58	mRNA	10875
Ju CKD TubInt	Lupus Nephritis vs. Healthy Living Donor	Disease vs. Control	over expression	< 0.0001	1.62	mRNA	10875
Ju CKD TubInt	Diabetic Nephropathy vs. Healthy Living Donor	Disease vs. Control	over expression	< 0.0001	2.17	mRNA	10875
Nakagawa CKD Kidney	Chronic Kidney Disease vs. Normal Kidney (Discovery Set)	Disease vs. Control	over expression	< 0.0001	1.65	mRNA	A_23_P42969
Nakagawa CKD Kidney	Chronic Kidney Disease vs. Normal Kidney (Validation Set)	Disease vs. Control	over expression	0.00700	2.86	mRNA	A_23_P42969
Neusser Hypertension Glom	Nephrosclerosis vs. Tumor Nephrectomy	Disease vs. Control	over expression	< 0.0001	2.17	mRNA	204834_at
Reich IgAN Glom	IgA Nephropathy vs. Healthy Living Donor	Disease vs. Control	over expression	< 0.0001	2.98	mRNA	204834_at
Schmid Diabetes TubInt	Diabetic Nephropathy vs. Control	Disease vs. Control	over expression	0.00030	2.96	mRNA	204834_at
Woroniecka Diabetes TubInt	Diabetic Nephropathy vs. Healthy Living Donor	Disease vs. Control	over expression	0.00016	2.83	mRNA	204834_at

Analyses for FGL2. Applied filters: Fold change: 1.5, Disease vs. Control Analyses, Glomeruli, Other Kidney Part, Tubulointerstitium, Human. Data was extracted on September 19th 2019 and updated on September 2nd 2021. A p-value < 0.05 was considered statistically significant.

Supplementary Data S1 – Members of the Nephrotic Syndrome Study Network (NEPTUNE)

NEPTUNE Enrolling Centers

Cleveland Clinic, Cleveland, OH: K Dell*, J Sedor**, B Martin#

Children's Hospital, Los Angeles, CA: K Lemley*, S Tang#

Children's Mercy Hospital, Kansas City, MO: T Srivastava*, K Markus#

Cohen Children's Hospital, New Hyde Park, NY: C Sethna*, S Vento #

Columbia University, New York, NY: P Canetta*, A Pradhan#

Emory University, Atlanta, GA: L Greenbaum*, C Wang**, E Yun#

Harbor-University of California Los Angeles Medical Center: S Adler*, J LaPage#

John H. Stroger Jr. Hospital of Cook County, Chicago, IL: A Athavale*, M Itteera

Johns Hopkins Medicine, Baltimore, MD: M Atkinson*, T Dell#

Mayo Clinic, Rochester, MN: F Fervenza*, M Hogan**, J Lieske*#

Montefiore Medical Center, Bronx, NY: F Kaskel*, M Ross*, P Flynn#

NIDDK Intramural, Bethesda MD: J Kopp*

New York University Medical Center, New York, NY: L Malaga-Dieguez*, O Zhdanova**, B Pace#

Stanford University, Stanford, CA: R Lafayette*, S Dave#

Temple University, Philadelphia, PA: I Lee*, S Quinn-Boyle#

University Health Network Toronto: H Reich *, M Hladunewich**, P Ling#, M Romano#

University of Miami, Miami, FL: A Fornoni*, C Bidot#

University of Michigan, Ann Arbor, MI: M Kretzler*, D Gipson*, A Williams#, C Klida#

University of North Carolina, Chapel Hill, NC: V Derebail*, K Gibson*, A Froment#, F Ochoa-Toro#

University of Pennsylvania, Philadelphia, PA: L Holzman*, K Meyers**, K Kallem#, A Swenson#

University of Texas Southwestern, Dallas, TX: K Sambandam*, K Aleman#, M Rogers#

University of Washington, Seattle, WA: A Jefferson*, S Hingorani**, K Tuttle**§, L Manahan #, E Pao#, K Kuykendall K§

Wake Forest University Baptist Health, Winston-Salem, NC: JJ Lin*, Stefanie Baker#

Data Analysis and Coordinating Center

M Kretzler*, L Barisoni**, C Gadegbeku**, B Gillespie**, D Gipson**, L Holzman**, L Mariani**, M Sampson**, J Sedor**, J Zee**, G Alter, H Desmond, S Eddy, D Fermin, M Larkina, S Li, S Li, CC Lienczewski, T Mainieri, R Scherr, A Smith, A Szymanski, A Williams.

Digital Pathology Committee

Carmen Avila-Casado (University Health Network, Toronto), Serena Bagnasco (Johns Hopkins University), Joseph Gaut (Washington University in St Louis), Stephen Hewitt (National Cancer Institute), Jeff Hodgin (University of Michigan), Kevin Lemley (Children's Hospital of Los Angeles), Laura Mariani (University of Michigan), Matthew Palmer (University of Pennsylvania), Avi Rosenberg (Johns Hopkins University), Virginie Royal (University of Montreal), David Thomas (University of Miami), Jarcy Zee (University of Pennsylvania) Co-Chairs: Laura Barisoni (Duke University) and Cynthia Nast (Cedar Sinai).

* Principal Investigator; ** Co-investigator; #Study Coordinator

§ Providence Medical Research Center, Spokane, WA
